# *Phyllanthus amarus* Schumach. and Thonn. and *Andrographis paniculata* (Burm. f.) Nees modulates enzymes associated with erectile dysfunction in Streptozotocin-induced diabetic male rats

**Published:** 2025

**Authors:** Ajiboye Oluwapelumi Micheal, Oyeleye Idowu Sunday, Adedayo Bukola Christiana, Abua Christopher Oshie, Adedeji Oluwadayomi Esther, Oboh Ganiyu

**Affiliations:** 1 *Functional Food and Nutraceutical Unit, Department of Biochemistry, Federal University of Technology, P.M.B. 704, Akure, Ondo State, Nigeria*; 2 *Department of Basic Sciences, School of Science and Technology, Babcock University, Ilishan-Remo, Ogun State, Nigeria*; 3 *Department of Biomedical Technology, Federal University of Technology, P.M.B. 704, Akure, Ondo State, Nigeria *

**Keywords:** Erectile dysfunction, Diabetes mellitus, Phyllanthus amarus, Andrographis paniculate, Endothelial dysfunction

## Abstract

**Objective:**

Erectile dysfunction (ED) is a prevalent complication among diabetic patients, and it is associated with oxidative stress, endothelial dysfunction, and diminished nitric oxide generation. This study investigates the therapeutic efficacy of alkaloid extracts from *Phyllanthus amarus* and *Andrographis paniculata* on biochemicals related to ED in diabetic rats.

**Materials and Methods:**

Male Wistar rats were divided into seven groups: non-diabetic control, untreated diabetic, standard drug-treated diabetic (5 mg/kg glibenclamide), and four extract-treated diabetic groups (5 and 50 mg/kg of each plant alkaloid extract). Diabetes was induced via intraperitoneal injection of streptozotocin (STZ). Treatments were administered orally, once daily, for a duration of 21 days.

**Results:**

Biochemical analysis demonstrated that diabetic rats had elevated phosphodiesterase-5 (PDE-5) and arginase activities and diminished antioxidant molecules. Treatment with plant extracts significantly inhibited PDE-5 and arginase activities, restored antioxidant enzymes (superoxide dismutase, catalase, glutathione-S-transferase, and reduced glutathione), and decreased malondialdehyde (MDA) and reactive oxygen species levels, indicating their potential to mitigate oxidative stress. The extracts had an inhibitory effect on acetylcholinesterase and butyrylcholinesterase activities, hence augmenting cholinergic signaling.

**Conclusion:**

Alkaloid extracts of *P. amarus* and *A. paniculata* may enhance nitric oxide levels in endothelial cells, inhibiting key enzymes and improving antioxidant status. Their potential to inhibit PDE-5 and arginase, alongside their antioxidant properties, suggests they may offer a safer alternative to conventional treatments for diabetic ED. These findings highlight their therapeutic potential as a holistic approach to managing the complex nature of diabetes-related ED.

## Introduction

Erectile dysfunction (ED) is a condition characterized by the inability to achieve or maintain a sustainable penile rigidity for successful sexual intercourse (S. I. Oyeleye, Adefegha, Dada, Okeke, & Oboh, 2019; Xiong et al., 2023). ED is becoming a prevalent condition in individuals with diabetes with approximately 40% affected at the age between 40 and 70 years old (Al-Oanzi, 2019; Cayetano-Alcaraz, Tharakan, Chen, Sofikitis, & Minhas, 2023; S. I. Oyeleye, Ojo, & Oboh, 2021). The prevalence of ED has increased from 5% at age 40 to 15% at age 70 (Oyelade, Jemilohun, & Aderibigbe, 2016). Studies have shown that the etiology of diabetes-linked ED is multifaceted, including old age, impaired arterial blood flow, damage to endothelial tissue, and nervous system disturbances (Akomolafe, Olasehinde, Ogunsuyi, Oyeleye, & Oboh, 2019; S. I. Oyeleye et al., 2021). Hyperglycemia-induced oxidative stress, inflammation, and endothelial dysfunction contribute to the development and progression of this debilitating condition (Bhamidipati et al., 2022; Nor et al., 2022). Oxidative stress exacerbates endothelial dysfunction, while endothelial damage feeds back to worsen oxidative stress (Idowu Sunday Oyeleye, Ojo, & Oboh, 2023). The combined effects impair vascular smooth muscle relaxation, reduce penile blood flow, and undermine erectile rigidity. Hyperglycemia in diabetes accelerates the production of reactive oxygen species and reduces the antioxidant defense mechanism, leading to a state of oxidative stress. ROS, including superoxide anions and hydrogen peroxide, damage cellular macromolecules such as lipids, proteins, and DNA (Olagunju, Oluwajuyitan, & Oyeleye, 2020). Oxidative insult is particularly detrimental to penile tissues where it diminishes endothelial function by depleting nitric oxide (NO) bioavailability (Singh, Yadav, Sharma, & Jin, 2021). Furthermore, oxidative stress promotes lipid peroxidation and malondialdehyde (MDA) production which correlate with vascular complications, including ED (Kaltsas, Zikopoulos, et al., 2024). 

Endothelial cells are a critical regulator of vascular tone and are highly susceptible to oxidative damage in diabetes. In diabetes, endothelial dysfunction arises due to reduce NO production, increased arginase activity, and pro-inflammatory state. Enzymes such as arginase and NO-Synthase (NOS) are pivotal in ED, as arginase catalyzes the conversion of L-arginine into urea and ornithine, whereas NOS transforms L-arginine into NO and citrulline to sustained sexual arousal and penile rigidity (Marzęta-Assas, Jacenik, & Zasłona, 2024). Both enzymes compete for the substrate L-arginine, inhibiting the sustained production of cGMP which is essential for maintaining and prolonging an erection, by facilitating smooth muscle relaxation, allowing increased blood flow to the corpus cavernosum. Elevated arginase activity in diabetes limits NO production and contributes to vascular dysfunction. Additionally, chronic hyperglycemia fosters inflammation, increasing cytokine production (e.g., Tumor Necrosis Factor Alpha (TNF-α) and Interleukin-6 (IL-6)) and further impairing endothelial cell function (Idowu Sunday Oyeleye, Ojo, Ajeigbe, & Oboh, 2024).

Phosphodiesterase-5 (PDE-5), an essential NO/cGMP signaling pathway enzyme, regulates smooth muscle cell contraction and erectile function. Inhibition of arginase and PDE-5 activity is vital in treating and managing ED (Panklai et al., 2023). Nonetheless, in diabetes-induced ED, addressing ED without adequate control of blood glucose levels may not provide a sustainable cure, since it only addresses the superficial symptoms. Conversely, managing diabetes through blood glucose reduction effectively addresses the underlying difficulties as well. 

Conventional treatments for ED and diabetes can be helpful, but they have important drawbacks. This shows that there is a need for different options. PDE-5 inhibitors like Sildenafil, Tadalafil, and Avanafil (Maleki-saghooni, Mirzaeii, Hosseinzadeh, Sadeghi, & Irani, 2018) and conventional antidiabetic medications (such as glibenclamide, acarbose, metformin, etc.) used for managing these disease conditions are however, associated with adverse effects and post-treatment experiences including nausea, diarrhea, abdominal pain and bloating, headache, flushing, nasal congestion, nasopharyngitis, and dyspepsia and priapism (Ajiboye, Ogunwenmo, Adewumi, & Mohanye, 2024; Chaudhury et al., 2017) Hence, therapeutic solutions from natural plants would be a better alternative for management of the complex disease conditions, since they are known to have minimal or no side effects (Ajiboye, Adewumi, et al., 2024; Fabiyi et al., 2024).

*Andrographis paniculata*, also known as the “King of bitters”, belongs to the Acanthaceae family (Murthy, Park, & Paek, 2021), and has been utilized as a traditional herbal remedy in Asian, Chinese, and African countries to treat several diseases including malaria fever, diarrhea, high blood pressure, cold and ulcers, among many others (Abdel-Hamid, Abass, Mohamed, & Muneam Hamid, 2018; Adedayo, Ajiboye, Oyeleye, Ojo, & Oboh, 2023; Hossain, Urbi, Sule, & Rahman, 2014). Extracts from *A. paniculata* have been reported to have potent antimalarial, antioxidant, neuroprotective, hepatoprotective, antiviral, and anticancer activities (Abhishek Niranjan, S. K. Tewari, & Lehri, 2010; Adedayo et al., 2023; Gupta, Mishra, & Ganju, 2016; J. R. Patel, Tripathi, Sharma, Chauhan, & Dixit, 2011). *Phyllanthus amarus* is one of the most common members of the Phyllanthaceae family, widely used in folk medicine, and found in subtropical regions (Ngo et al., 2020; Rehman et al., 2021). It has gained global recognition for its hepatoprotective, antihypertensive, antimicrobial, and antibacterial properties. Phytochemical studies have reported *P. amarus *to contain several bioactive compounds such as alkaloids, lignans, flavonoids, triterpenes, polyphenols, and tannin (J. R. Patel et al., 2011)

The study sought to investigate the therapeutic efficacy of alkaloid extracts from *Phyllanthus amarus* and *Andrographis paniculata* leaves on biochemicals (PDE-5, arginase, malondialdehyde, reactive oxygen species, acetylcholinesterase, butyrylcholinesterase, catalase, superoxide dismutase, glutathione-S-transferase, reduced glutathione) related to ED in streptozotocin (STZ)-induced diabetic rats.

## Materials and Methods

### Chemical and reagent

All chemicals were obtained from Sigma Chemicals (St. Louis, USA). All other substances including reagents were of analytical grade except where otherwise mentioned. 

### Sample collection and alkaloid preparation

Fresh leaves of *A. paniculata* and *P. amarus* were harvested from the botanical garden of the Federal University of Technology Akure (FUTA). Plant samples were identified and authenticated by the plant curator at the FUTA herbarium section (Voucher number: *P. amarus* - 17737 and *A. paniculata* – 17738, respectively). The leaves collected were air-dried under shade at room temperature, blended into fine powder, and stored for further use. Crude alkaloid was extracted from the plant's powdered samples following the procedure described by Ademiluyi, Ogunsuyi, Oboh, and Agbebi (2016). The percentage yields of the crude alkaloid extract were 5.22% and 5.95% for *A. paniculata* and *P. amarus*, respectively. The formula below was used to calculate percentage yields of the crude alkaloid extract:

$\mathrm{\% Crude Alkaloid}=\frac{\mathrm{mass of crude alkaloid extract}}{\mathrm{mass of initial dry plant}}\times \mathrm{100}$

### Animals handling

Five-week-old male Wistar albino rats of weight ranging between 110 and 125 g were purchased from the Department of Animal Production and Health, Ladoke Akintola University of Technology, Ogbomoso. All experimental animals were kept for a 14-day acclimatization period, in a well-ventilated cage (12 hr light/dark cycle) at 25℃ ± 2℃, with access to water and standard pellets *ad libitum*. An ethical approval guided by the guidelines for the Care and Use of Laboratory Animals as stated by the US National Institutes of Health (NIH) and European Union recommendation for animal experiments (Directive 2010/63/EU), was obtained from the ethical committee of the Centre for Research and Development (CERAD), FUTA, Ondo state.

### Induction of diabetes and animal grouping

The male Wistar rats were administered a single intraperitoneal injection of 50 mg/kg STZ diluted in 0.1 M citrate buffer. The non-diabetic control group only received the same amount of citrate buffer as the diabetic group. Blood samples were collected from the tail vein 48 hr after the injection to determine blood glucose levels, and rats with plasma glucose levels greater than 200 mg/dl were considered diabetic. Thereafter, the rats were randomly divided into seven groups (n = 6), where Group I normal rats served as the normal control. Group II-VII were diabetic rats, however Group II received no treatment while Group III was treated with a standard conventional drug (glibenclamide). Group IV and V were treated with 5 and 50 mg/kg alkaloid extract from *P. amarus*, respectively; while Group VI and VII were treated with an alkaloid extract from *A. paniculata*, at 5 and 50 mg/kg, respectively. The treatment doses were according to the previous study of Adedayo et al. (2023)

### Preparation of tissue homogenate

The treatment lasted 21 days (Sachdewa & Khemani, 2003), followed by overnight fasting for about 12 hr before euthanizing the animal via cervical dislocation. The penile tissues were carefully excised, washed with a cold saline solution, and weighed. The tissue was homogenized with 0.1 M phosphate buffer (pH 7.4) in a Teflon glass homogenizer and centrifuged for about 10 min at 3000 ×g to obtain a clear supernatant. The obtained supernatant was stored at -4℃ for further biochemical analysis.

### Biochemical assays

The stored clear supernatant was used to assay for related biochemical analysis including, PDE-5 (Oboh, Ademiluyi, Oyeleye, Olasehinde, & Boligon, 2017), arginase (Kaysen & Strecker, 1973), acetylcholinesterase (AChE), and butyrylcholinesterase (BChE) (George L. Ellman, Courtney, Andres, & Featherstone, 1961). The level of free radicals produced was also assayed via malondialdehyde (MDA) quantification (Ohkawa, Ohishi, & Yagi, 1979), and reactive oxygen species (ROS) according to the method described by Adedayo et al. (2023). Antioxidant status was also evaluated by determining the activities of enzymes including superoxide dismutase (SOD), catalase (CAT), and glutathione -S- transferase (GST) following the method of Akinyemi, Oboh, Ogunsuyi, Abolaji, and Udofia (2017); reduced glutathione (GSH) content was examined by the procedure described by G. L. Ellman (1959), as reported by Ogunsuyi, Ademiluyi, Oboh, Oyeleye, and Dada (2018) 

### Data analysis

All data were analyzed with one-way ANOVA using GraphPad Prism version 8 software and expressed as the means ± standard error of the mean (SEM); followed by a Tukey’s post-hoc test where p<0.05 represents a significant difference.

## Results

The result presented in Figure 1 represents the effect of the alkaloid extracts on the PDE-5 activity in the experimental rat’s penile tissue. The results revealed that diabetes caused a significant (p<0.05) increase in PDE-5 activity compared with the normal control. The result also showed that PDE-5 activity was reduced in the penile tissue of rats treated with the extract when compared to untreated diabetic rats. The therapeutic effect exhibited a dose-dependent pattern, showing no significant difference in the group treated with 50 mg/kg of *A. paniculata*, while a significant difference (p<0.05) was observed in the group treated with 5 mg/kg compared to the normal control. Furthermore, there was a competing efficacy between the glibenclamide and 50 mg/kg *A. paniculata *treatment groups, since both showed no significant difference with the normal control rats.

According to Figure 2, the effect of the extracts on arginase activity in the penile tissue of the experimental rats was revealed. Arginase activity was significantly higher in the untreated diabetic rats compared to the normal control. However, treatment with alkaloid extracts significantly reduced the activity of the arginase enzyme when compared with the STZ-induced untreated group. More so, there was a significant (p<0.05) difference observed in the 5 mg/kg of both plant extract treatment group when compared with the normal control. 

**Figure:**
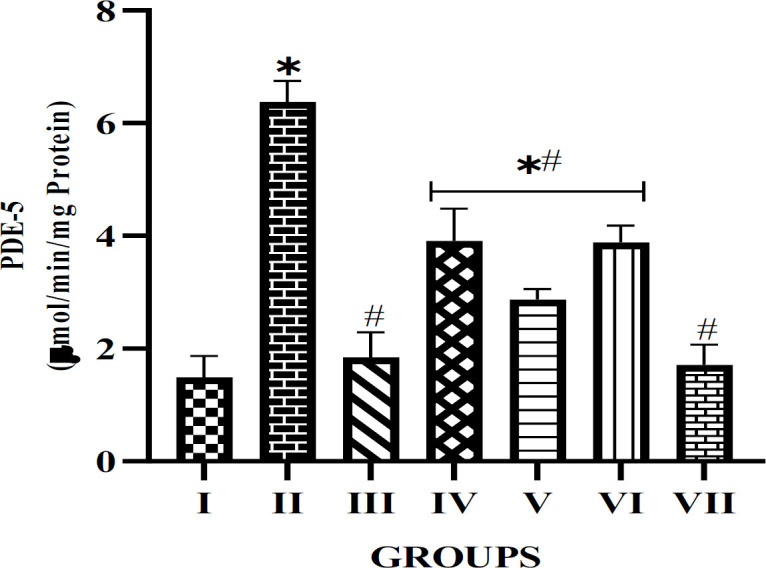


**Figure:**
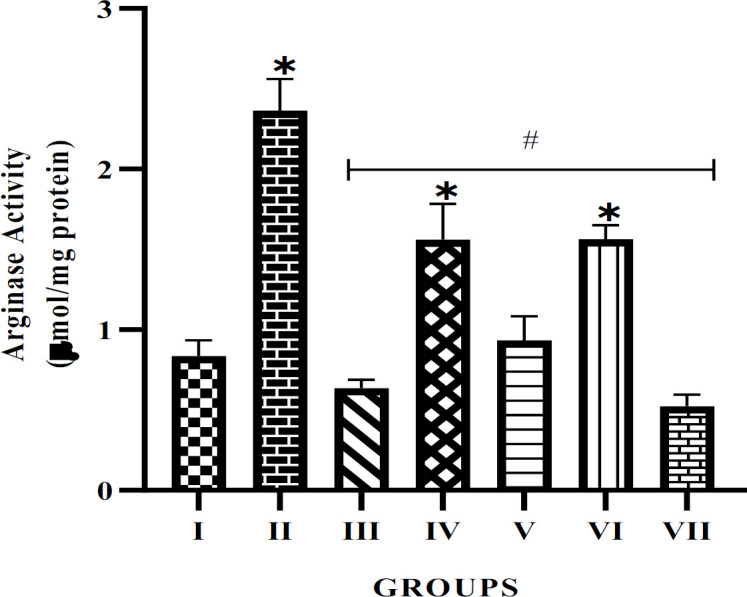


The result of the effect of the alkaloid extracts on AChE and BChE activities (Figure 3a and 3b) shows that both extracts have cholinesterase inhibitory effects, as the activities of both enzymes were reduced compared to the STZ-untreated group. Additionally, there was no significant difference observed in the 50 mg/kg treatment doses of both plant extract and the 5 mg/kg of *P. amarus* compared with the normal control group. As observed in the result, the AChE inhibitory effect of the alkaloid extract at higher doses is compared to that of the standard drug. A similar trend was also observed in the BChE activity. A significant (p<0.05) difference was observed in the 5 mg/kg treatment groups of both extracts when compared with normal control, and no significant difference in the 50 mg/kg extract and standard drug treatment groups compared to the STZ-induced untreated group was observed.

**Figure:**
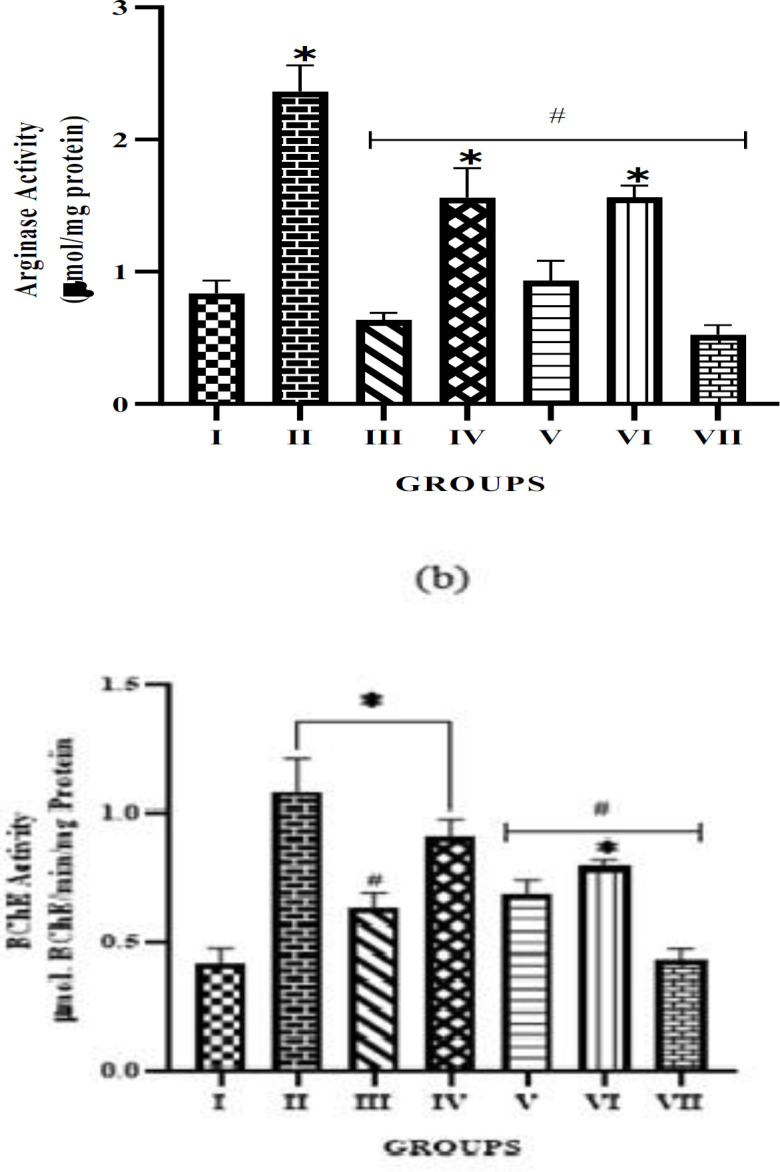


The free radical scavenging ability of alkaloid extract from *P. amarus *and *A. paniculata* leaves was also investigated. The results (Figures 4 and 5) show that diabetes caused an increased production of free radicals as observed in the untreated diabetic rats. However, both plants' alkaloid extracts have shown a significant free radical scavenging ability by reducing the level of production of MDA and ROS, particularly in the 50 mg/kg extract treatment groups when compared with the STZ-induced untreated group. Interestingly, the result showed a competing free radical scavenging ability between the 50 mg/kg and glibenclamide treatment group, with both showing no significant difference compared with the normal control group. 

**Figure:**
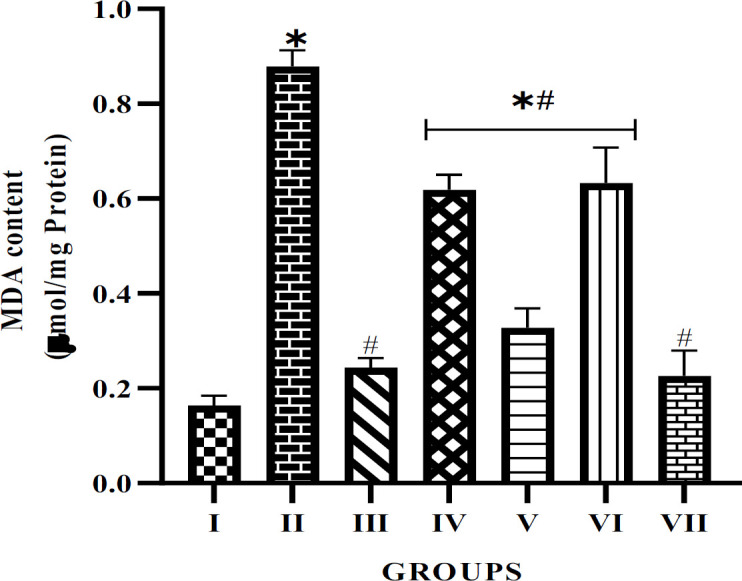


**Figure:**
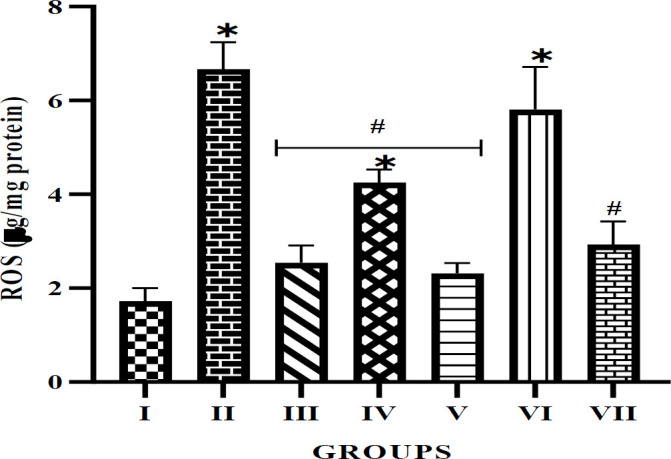


Diabetes-linked ED in the experimental rats caused a significantly decreased activity of the antioxidant enzymes, evidenced by the significant (p<0.05) difference observed in the antioxidant status of the STZ-induced untreated group, compared to the normal control. The results after treatment with the alkaloid extract from *P. amarus *and *A. paniculata *leaves improved antioxidant status, dose-dependently, as well as the glibenclamide treatment group. This was ascertained by the significant (p<0.05) increased activity of SOD, CAT, and GST activities, and GSH levels (Figure 6). 

**Figure:**
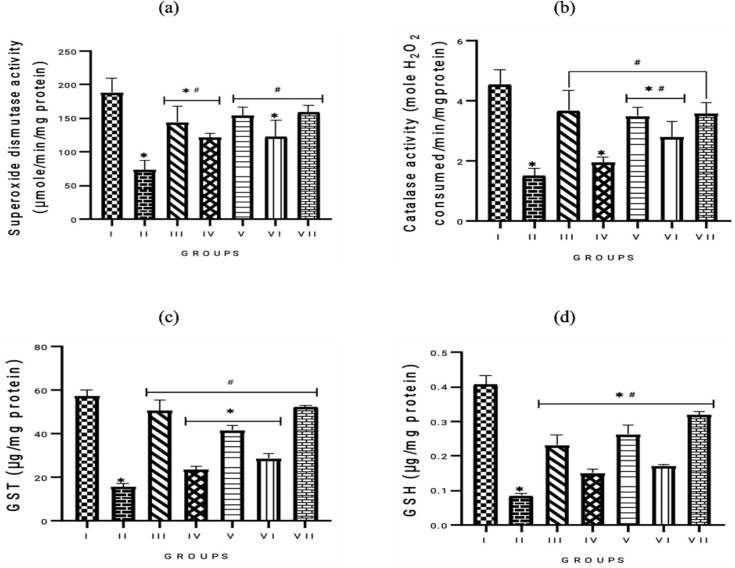


## Discussion

The pharmacotherapeutic effects of the studied plants have been recognized globally, including their anti-inflammatory, analgesic, antidiabetic, neuroprotective, and anti-cancer properties. There has been an increasing prevalence of ED, particularly among diabetic patients, associated with several factors including age, oxidative stress, neuronal disorder, endothelial tissue damage, or impairment of arterial blood flow (Kaltsas, Zachariou, et al., 2024; Kamenov, 2015). The dysregulation of PDE-5, known for its involvement in the relaxation of vascular smooth muscle, in diabetes has been implicated in several complications including ED and cardiovascular issues (Blanco-Rivero & Xavier, 2020; Koka & Kukreja, 2014). A significant increase in PDE-5 activity as a result of the diabetes condition was observed in the experimental animal, evident in the untreated diabetic rats. Previous studies have reported that persistent hyperglycemia level is linked to the upregulation of the PDE-5 activity in diabetic models (Nobili et al., 2021; Nwagwe, Adefegha, & Oboh, 2024). The alkaloid extract from the studied plants in this study, reduced PDE-5 enzyme activity in the penile tissue of extract-treated diabetic rats, thus, confirming its ED therapeutic potential. Previous findings by Omojokun, Famurewa, Jaiyeoba, Oboh, and Agbebi (2019) and Okeke, Adefegha, Oyeleye, and Oboh (2018) support the study finding that alkaloid-rich plant extracts possess PDE-5 inhibitory potential. The dose-dependent responses, with the lack of significant difference in the 50 mg/kg *A. paniculata *treatment group but a notable difference in the 5 mg/kg treatment group compared with the normal control, underscores the potency of the plant extract at the higher dosage. Natural compounds sometimes exhibit a threshold dose for optimal efficacy, which may explain the better outcomes observed at higher concentrations.

Nitric oxide (NO) production is crucial for vascular tone and erectile function, and arginase plays a role in regulating it (Förstermann & Sessa, 2012). Increased arginase activity can lead to impaired NO production, causing endothelial tissue damage, especially in conditions like diabetes. Studies have shown that diabetes can induce arginase activity, resulting in reduced NO bioavailability (C. Patel et al., 2013). Alkaloid extracts from *P. amarus* and *A. paniculata* have been shown to significantly reduce arginase activity, which could cause increased availability of L-arginine for NO production while improving endothelial function and mitigating the effects of diabetes on erectile physiology. The decrease in arginase activity suggests that plant compounds have therapeutic potential in restoring vascular health, potentially treating ED in diabetic patients.

AChE and BChE are enzymes that degrade cholinergic neurotransmitters, essential for facilitating smooth muscle relaxation and erectile function (Ojo et al., 2019). Endothelial dysfunction can result from cholinergic signaling impairment in diabetic conditions caused by hyperglycemia and oxidative stress. Alkaloid extracts from *P. amarus* and *A. paniculata* significantly inhibited the activities of AChE and BChE, indicating that these plants may enhance cholinergic signaling and improve erectile function in diabetic rats. The substantial cholinesterase-inhibiting properties suggest that the plant extracts may be just as effective in comparison to conventional anticholinesterase therapies. 

The plant extracts also decrease the levels of MDA and ROS in the penile tissues of diabetic rats. These biomolecules are indicators of lipid peroxidation and contribute to oxidative stress, which is a significant factor in the development of vascular complications in diabetes, including ED (Saad, Soliman, Mahmoud, Moneim, & Zaky, 2022). The decrease in MDA and ROS levels in the extract-treated diabetic rats suggests that *P. amarus* and *A. paniculata* exhibit potent antioxidant activities, possibly aiding in their protective effects against oxidative stress-related damage in diabetic tissues. Considering the similarity in free radical scavenging activities between the extract treatments and the glibenclamide-treated group, these plant extracts were as effective as conventional antidiabetic drugs in mitigating oxidative stress.

The study found that alkaloid extracts from *P. amarus* and *A. paniculata* improved antioxidant status in diabetic rats. Oxidative stress is crucial for the development of diabetes and related complications, such as ED. Diabetes impairs antioxidant defense systems, leading to the formation of ROS and oxidative tissue damage. The reduction in antioxidant enzyme activity including SOD, CAT, GST, and GSH levels, in the untreated diabetic rats, indicates oxidative stress associated with diabetic conditions. However, the enhancement in antioxidant enzyme activities in rats administered with alkaloid extracts suggests the extracts' potential to rejuvenate the antioxidant defense system by mitigating oxidative stress. The bioactive components in these plants exhibit potent antioxidant activities, neutralizing free radicals and enhancing endogenous antioxidant enzymes. Restoring antioxidant enzyme activity in diabetic individuals is essential for preventing tissue damage, enhancing vascular health, and mitigating ED.

The studied plants have demonstrated significant therapeutic benefits owing to their rich alkaloid constituent. Alkaloid constituents found in *P. amarus* and *A. paniculata* have been reported to effectively mitigate diabetic-linked ED by targeting oxidative stress, and impaired nitric oxide signaling. Phyllanthin and hypophyllanthin, which are found in *P. amarus*, have exhibited strong antioxidant properties that neutralize ROS and lessen oxidative damage to endothelial cells (Wan-Saidin et al., 2023). Andrographolide, found in *A. paniculata*, is a diterpenoid lactone that resembles an alkaloid and is well-known for its anti-inflammatory and antioxidant qualities (Owoade, Alausa, Adetutu1, Olorunnisola, & Owoade, 2021). It decreases oxidative stress markers, increases the activity of endogenous antioxidant enzymes, and inhibits PDE-5 (Hoppe, 2011). Neoandrographolide is another compound that complements these actions by reducing lipid peroxidation and strengthening antioxidant defenses, protecting against vascular and neural damage (Messire, Serreau, & Berteina-Raboin, 2023).

The findings from this study suggest that the therapeutic efficacy of these plant extracts is dose-dependent, with higher concentrations delivering more pronounced health benefits. This aligns with the pharmacological principle that certain bioactive compounds exhibit threshold doses for optimal activity. However, the lack of significant adverse effects at higher doses supports their safety profile, making them viable candidates for further clinical exploration. 

The alkaloid extracts from *P. amarus* and *A. paniculata* exhibit considerable therapeutic potential by inhibiting PDE-5, arginase, AChE, and BChE activities, enhancing NO bioavailability and consequently improving vascular health. The substantial antioxidant effects of the extracts are demonstrated by reduced oxidative stress indicators and enhanced antioxidant enzyme activity. Although the current research shows that the alkaloid extracts are effective in modulating biochemical markers related to ED in the penile tissue of diabetic rats, more investigation is required to examine the long-term effects of these extracts on erectile function and overall health in diabetic patients. Sexual behavior should also be investigated to affirm these claims.

## Abbreviations

AChE – Acetylcholinesterase

BChE – Butyrylcholinesterase

CAT – Catalase

CERAD – Center for Research and Development

ED – Erectile Dysfunction

EU – European Union

FUTA – Federal University of Technology Akure

GSH – Reduced Glutathione

GST – Glutathione -S- Transferase

MDA – Malondialdehyde

NIH – National Institutes of Health

NO – Nitric Oxide

NOS – Nitric Oxide Synthase

PDE-5 – Phosphodiesterase-5

ROS – Reactive Oxygen Species

SOD – Superoxide Dismutase

STZ – Streptozotocin

TNF-α – Tumor Necrosis Factor Alpha

IL-6 – Interleukin-6
